# Patterns of Antihypertensive Medication Use in the First 2 Years Post Partum

**DOI:** 10.1001/jamanetworkopen.2024.26394

**Published:** 2024-08-07

**Authors:** Frederikke Lihme, Saima Basit, Baskaran Thilaganathan, Heather A. Boyd

**Affiliations:** 1 Department of Epidemiology Research, Statens Serum Institut, Copenhagen, Denmark; 2 Fetal Medicine Unit, St George’s Hospital, University of London, London, United Kingdom

## Abstract

**Importance:**

Women who had a hypertensive disorder of pregnancy (HDP) have a well-documented risk of chronic hypertension within a few years of delivery, but management of postpartum hypertension among these women remains inconsistent.

**Objective:**

To assess the incidence of initiation of antihypertensive medication use in the first 2 years after delivery by HDP status and antenatal antihypertensive medication use.

**Design, Setting, and Participants:**

This Danish register-based cohort study used data from women with at least 1 pregnancy lasting 20 or more gestational weeks (only the first pregnancy in the period was considered) who delivered from January 1, 1995, to December 31, 2018. Statistical analysis was conducted from October 2022 to September 2023.

**Exposure:**

Hypertensive disorders of pregnancy.

**Main Outcomes and Measures:**

Cumulative incidences and hazard ratios of initiating antihypertensive medication use within 2 years post partum (5 postpartum time intervals) by HDP status and antenatal medication use.

**Results:**

The cohort included 784 782 women, of whom 36 900 (4.7% [95% CI, 4.7%-4.8%]) had an HDP (HDP: median age at delivery, 29.1 years [IQR, 26.1-32.7 years]; no HDP: median age at delivery, 29.0 years [IQR, 25.9-32.3 years]). The 2-year cumulative incidence of initiating postpartum antihypertensive treatment ranged from 1.8% (95% CI, 1.8%-1.8%) among women who had not had HDPs to 44.1% (95% CI, 40.0%-48.2%) among women with severe preeclampsia who required antihypertensive medication during pregnancy. Most women who required postpartum antihypertensive medication after an HDP initiated use within 3 months of delivery (severe preeclampsia, 86.6% [95% CI, 84.6%-89.4%]; preeclampsia, 75.3% [95% CI, 73.8%-76.2%]; and gestational hypertension, 75.1% [95% CI, 72.9%-77.1%]). However, 13.4% (95% CI, 11.9%-14.1%) of women with severe preeclampsia, 24.7.% (95% CI, 24.0%-26.0%) of women with preeclampsia, 24.9% (95% CI, 22.5%-27.5%) of women with gestational hypertension, and 76.7% (95% CI, 76.3%-77.1%) of those without an HDP first filled a prescription for antihypertensive medication more than 3 months after delivery. Women with gestational hypertension had the highest rate of initiating medication after more than 1 year post partum, with 11.6% (95% CI, 10.0%-13.2%) starting treatment after this period. Among women who filled a prescription in the first 3 months post partum, up to 55.9% (95% CI, 46.2%-66.1%) required further prescriptions more than 3 months post partum, depending on HDP status and antenatal medication use.

**Conclusions and Relevance:**

In this cohort study of postpartum women, the incidence of initiation of postnatal antihypertensive medication use varied by HDP status, HDP severity, and antenatal antihypertensive medication use. Up to 24.9% of women initiated antihypertensive medication use more than 3 months after an HDP, with up to 11.6% initiating treatment after 1 year. Routine postpartum blood pressure monitoring might prevent diagnostic delays in initiation of antihypertensive medication use and improve cardiovascular disease prevention among women.

## Key Points

**Question:**

How do patterns of initiation of antihypertensive medication use within 2 years of delivery compare among women with or without hypertensive disorders of pregnancy (HDP)?

**Findings:**

This cohort study of 784 782 women found that postnatal antihypertensive medication use increased with HDP severity, early diagnosis, and antenatal medication use. Up to 44.1% of women with HDPs and 1.8% of women with normotensive pregnancies initiated medication use within 2 years of delivery, with 24.9% of women with HDPs and 76.7% of the women with normotensive pregnancies first initiating use more than 3 months post partum.

**Meaning:**

This study suggests that routine, systematic postpartum blood pressure monitoring might prevent the observed delays in initiation of antihypertensive medication use, thereby improving cardiovascular disease prevention among women.

## Teaser

This register-based cohort study assesses the incidence of initiation of antihypertensive medication use in the 2 years post partum among women with or without hypertensive disorders of pregnancy.

## Introduction

In Denmark, hypertensive disorders of pregnancy (HDPs) affect approximately 2% to 5% of pregnancies.^1^ Although women who had HDPs have postpartum risks of chronic hypertension that are up to 10 times those observed among women with no history of HDPs and substantially increased long-term risks of cardiovascular disease and renal dysfunction,^2,3,4,5,6,7,8^ postpartum management of women who had HDPs is inconsistent and fragmented.

Persistent high blood pressure 6 weeks after a pregnancy complicated by an HDP is associated with later chronic hypertension,^9^ which increases the risk of cardiovascular disease.^10,11^ However, randomized clinical trials have shown that strict blood pressure control in the immediate postpartum period produces reductions in diastolic blood pressure that persist 3 to 4 years post partum,^12,13^ suggesting that, to mitigate cardiovascular disease risk and improve long-term outcomes among women who had HDPs, women who continue to have hypertension after delivery should immediately initiate treatment with antihypertensive medication and be strictly managed until they no longer have hypertension.^9,10,11,14^

Understanding existing postpartum care patterns would help health care systems to determine what efforts are needed to deliver appropriate postpartum follow-up to women who had HDPs and address persistent postpartum hypertension as early as possible, but data on current practices are lacking. Therefore, in a cohort of more than 780 000 women with pregnancies in Denmark from 1995 to 2018, we investigated the timing, cumulative incidence, and relative risk of initiating antihypertensive medication use within 2 years of delivery among women with or without an HDP.

## Methods

### Study Cohort

Using the Danish Civil Registration System, the Medical Birth Register, and the National Patient Register,^15,16,17^ we identified all Danish residents with a pregnancy lasting at least 20 weeks who delivered in Denmark (live birth or stillbirth) from January 1, 1995, to December 31, 2018. For women with more than 1 pregnancy in this period, we considered only the first pregnancy to ensure that medication use in the postpartum period was associated with events in the pregnancy immediately before and not with events occurring in an earlier pregnancy. We excluded from the cohort women with major congenital defects as defined by EUROCAT (European Surveillance of Congenital Anomalies),^18^ women with cardiovascular disease registered before pregnancy, and women with hypertension diagnosed or treated before week 20 of the study pregnancy (see eAppendix in Supplement 1 for definitions). This study was approved by Statens Serum Institut’s Department of Data Protection and Information Security and registered with the Danish Data Protection Agency. Under Danish law, neither informed consent nor ethics committee approval is required for strictly register-based studies. The analyses and presentation of results followed the Strengthening the Reporting of Observational Studies in Epidemiology (STROBE) reporting guideline for cohort studies.

### HDPs and Postpartum Initiation of Antihypertensive Medication Use

Using the National Patient Register, we considered women to have had an HDP if they were registered with a diagnosis of gestational hypertension (GH), preeclampsia without severe features (hereafter, *preeclampsia*), or preeclampsia with severe features, including the HELLP (hemolysis, elevated liver enzymes and low platelets) syndrome and eclampsia (hereafter, *severe preeclampsia*) (see eAppendix in Supplement 1 for details).

We considered a woman to have initiated postpartum use of antihypertensive medication if the National Prescription Registry^19^ showed that she filled at least 1 prescription for a β-blocking agent, a calcium channel blocker, an agent acting on the renin-angiotensin system (hereafter, *renin-angiotensin system blockers*), a diuretic, or another specified antihypertensive agent in the postpartum period (see Appendix in Supplement 1 for Anatomic Therapeutic Chemical codes). Filled prescriptions are registered automatically via the Danish personal identification number,^16^ ensuring that registration of filled prescriptions is virtually complete.

### Statistical Analysis

Statistical analysis was conducted from October 2022 to September 2023. We estimated cumulative incidences of postpartum initiation of antihypertensive medication use and hazard ratios (HRs) for initiation of medication use. In both types of analyses, we followed up with women from their date of delivery until the first of the following: (1) date of filling a prescription for antihypertensive medication, (2) 2 years had elapsed since delivery, (3) week 20 of their second pregnancy in the study period, (4) death, (5) emigration, (6) registration as “missing” in the Danish Civil Registration System,^16^ or (7) December 31, 2018 (end of follow-up).

When estimating the 2-year cumulative incidence of postpartum initiation of antihypertensive medication use, we stratified the analyses by type of HDP (none, GH, preeclampsia, or severe preeclampsia) and whether antenatal antihypertensive medication was prescribed after 20 weeks’ gestation, presumably to counter new-onset hypertension in pregnancy. In addition, we further categorized women according to whether they delivered preterm (<37 gestational weeks) and whether their infant was small for gestational age (birth weight for gestational age and sex <10th percentile), and determined the proportion of women in each subgroup who initiated use of antihypertensive medication within 2 years of delivery. Among women who filled a prescription within 3 weeks of delivery, we then estimated the cumulative incidence of filling another prescription more than 3 months post partum to illustrate the proportion of women whose post-HDP hypertension did not resolve after an initial course of treatment. In this analysis, we followed up with the women from 3 months post partum until the first of the outcomes listed.

Using Cox proportional hazards regression with maternal age as the underlying time scale, we estimated HRs for postpartum initiation of antihypertensive medication use, comparing women with and women without HDPs. Because checks of the proportional hazards assumption showed that it did not hold over the entire 2-year follow-up period, we present HRs stratified by intervals of time elapsed since delivery (<3 months, 3-5 months, 6-11 months, 12-17 months, and 18-23 months), within which the HRs were piecewise constant. We adjusted our analyses for maternal birth year to accommodate changes in HDP definitions and treatment over time. Finally, we examined the maximum number of different medications used in any 1-month period during the first 2 postpartum years (or up to 20 weeks’ gestation in a next pregnancy) and investigated whether the class of medication first prescribed varied by time elapsed between delivery and initiation of treatment and HDP status. All *P* values were from 2-sided tests and results were deemed statistically significant at *P* < .05.

## Results

The study cohort included 784 782 women with at least 1 pregnancy from 1995 to 2018, of whom 36 900 (4.7% [95% CI, 4.7%-4.8%]) had an HDP (HDP: median age at delivery, 29.1 years [IQR, 26.1-32.7 years]; no HDP: median age at delivery, 29.0 years [IQR, 25.9-32.3 years]). Cohort characteristics delivery are shown by type of HDP in Table 1. Information on maternal birth year, age at delivery, and date of delivery was available for the entire cohort, as registration of these key variables is virtually complete for Danish residents. Information on gestational age at delivery was missing for 1.6% of the cohort, and information on birth weight was missing for 0.8% of the cohort, such that classification of offspring birth weight for gestational age was missing for 2.4% of women. In the last half of pregnancy, 3862 women (0.5% [95% CI, 0.5%-0.5%]) filled 1 or more outpatient prescriptions for antihypertensive medication.

**Table 1.** Cohort Characteristics at Delivery After the First Pregnancy of at Least 20 Weeks’ Duration by Type of Hypertensive Disorder of Pregnancy, 1995-2018, Denmark

Maternal characteristic	No. (%)			
	No hypertensive disorder of pregnancy (n = 747 882)	Gestational hypertension (n = 8772)	Preeclampsia (n = 20 051)	Severe preeclampsia (n = 8077)
Maternal age, y				
<25	142 708 (19.1)	1290 (14.7)	3954 (19.7)	1439 (17.8)
25-29	294 713 (39.4)	3310 (37.7)	8009 (39.9)	3061 (37.9)
30-34	217 775 (29.1)	2688 (30.6)	5470 (27.3)	2382 (29.5)
35-39	78 106 (10.4)	1179 (13.4)	2130 (10.6)	952 (11.8)
≥40	14 580 (2.0)	305 (3.5)	488 (2.4)	243 (3.0)
Maternal BMI^a^				
<18.5	17 615 (2.4)	115 (1.3)	247 (1.2)	169 (2.1)
18.5-24	234 377 (31.4)	2793 (31.8)	5268 (26.7)	2756 (34.1)
25-29	68 773 (9.2)	1495 (17.0)	2872 (14.3)	1080 (13.4)
30-34	24 027 (3.2)	847 (9.7)	1411 (7.0)	476 (5.9)
35-39	7867 (1.1)	394 (4.5)	645 (3.2)	193 (2.4)
≥40	3458 (0.5)	242 (2.8)	337 (1.7)	117 (1.5)
Not available	391 765 (52.4)	2885 (32.9)	9171 (45.7)	3286 (40.7)
Gestational age at delivery, wk				
<28	2781 (0.4)	17 (0.2)	29 (0.1)	278 (3.4)
28-36	42 287 (5.8)	526 (6.0)	2090 (10.4)	3617 (44.8)
≥37	690 587 (92.3)	8189 (93.4)	17 772 (88.6)	4126 (51.1)
Not available	12 227 (1.6)	40 (0.5)	161 (0.8)	56 (0.7)
Birth weight				
Appropriate for gestational age	571 491 (76.4)	6261(71.4)	13 466 (67.2)	4123 (51.1)
Small for gestational age^b^	96 402 (12.9)	1582 (18.0)	4137 (20.6)	3347 (41.4)
Large for gestational age^c^	61 591 (8.2)	830 (9.5)	2120 (10.6)	432 (5.4)
Not available	18 398 (2.4)	99 (1.1)	328 (1.6)	175 (2.1)

Abbreviation: BMI, body mass index (calculated as weight in kilograms divided by height in meters squared).

^a^ Available from 2003.

^b^ Birth weight less than the 10th percentile for gestational age and sex.

^c^ Birth weight higher than the 10th percentile for gestational age and sex.

### Cumulative Incidence of Initiating Postpartum Antihypertensive Medication Use

Overall, 17 897 women (2.3% [95% CI, 2.3%-2.3%]) initiated use of antihypertensive medication during the first 2 years post partum. For 161 480 women (20.6% [95% CI, 20.5%-20.7%]), follow-up ended without medication use but before 2 years had elapsed; the most common reason for an early end to follow-up was a new pregnancy (150 476 of 161 480 [93.2%]), followed by emigration (9909 of 161 480 [6.1%]), death (916 of 161 480 [0.6%]), and disappearance from the registers (179 of 161 480 [0.1%]).

**Figure 1.**
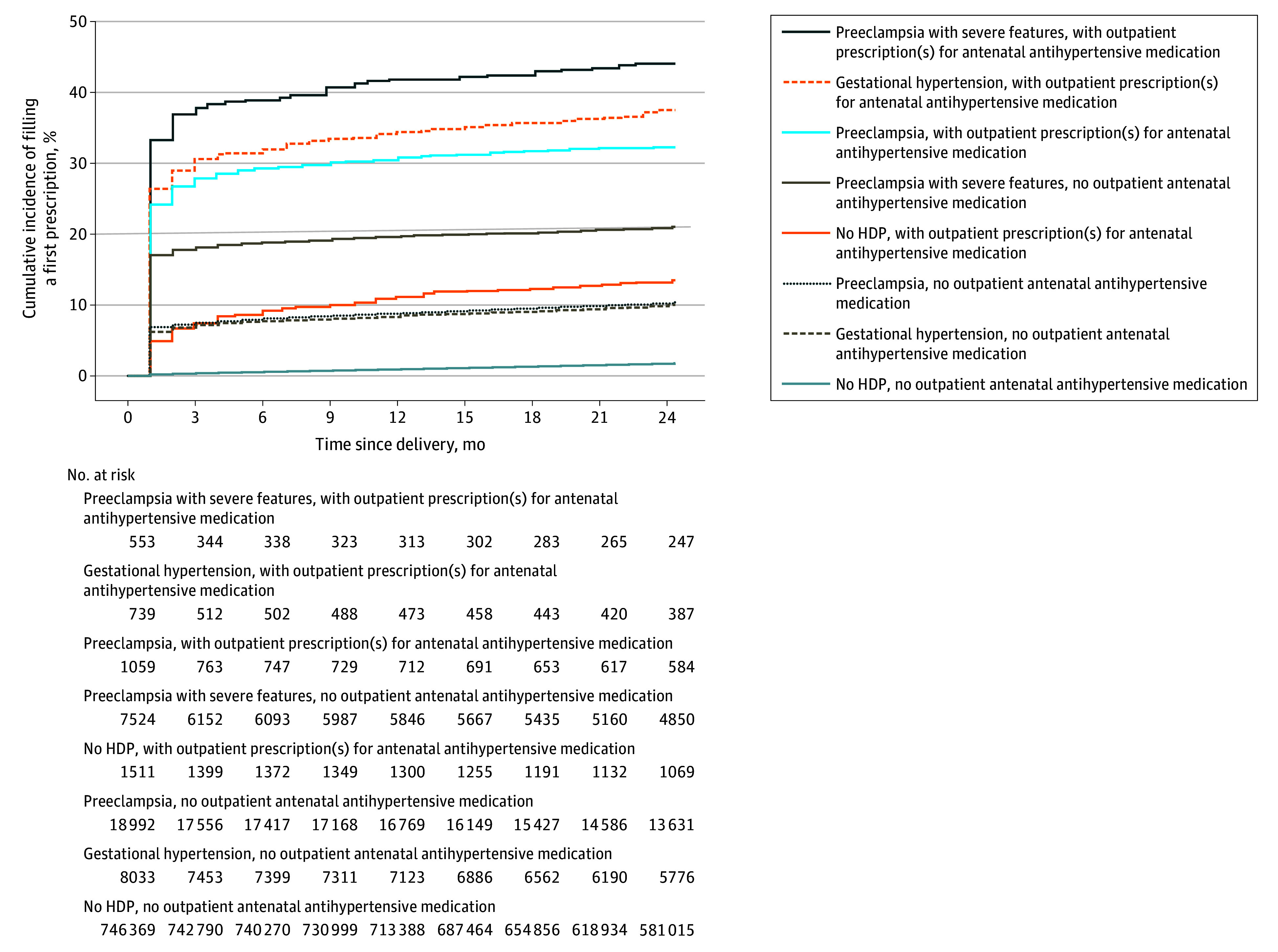
Two-Year Cumulative Incidences of Initiating Postpartum Antihypertensive Medication Use, by Type of Hypertensive Disorder of Pregnancy (HDP) and Whether or Not Outpatient Prescriptions for Antihypertensive Medication Were Filled During Pregnancy, After a Woman’s First Delivery in the Study Period, 1995-2018, Denmark Preeclampsia with severe features includes eclampsia or the HELLP (hemolysis, elevated liver enzymes and low platelets) syndrome. A lack of antenatal outpatient prescriptions for antihypertensive medication does not preclude the receipt of inpatient or outpatient clinic–based treatment with antihypertensive medication during pregnancy, as medications dispensed by hospital pharmacies are not registered in the Danish National Prescription Register (which contains information only on prescriptions filled at community pharmacies).

Figure 1 shows the cumulative incidence of initiating postpartum use of antihypertensive medication by HDP status and use of antenatal antihypertensive medication. Most women requiring antihypertensive medication post partum initiated use within 3 months of delivery, during which time the cumulative incidence of filling a first prescription ranged from 7.5% (95% CI, 7.0%-8.0%) to 38.3% (95% CI, 34.4%-42.5%) after an HDP (eTable 1 in Supplement 1). Women with HDPs who required antenatal antihypertensive medication had the highest 2-year incidences of postpartum medication use (after severe preeclampsia, 44.1% [95% CI, 40.0%-48.2%]; after GH, 37.5% [95% CI, 34.1%-41.1%]; and after preeclampsia, 32.2% [95% CI, 29.5%-35.1%] after severe preeclampsia, GH, and preeclampsia, respectively).

Women who delivered preterm were more likely to initiate antihypertensive medication use post partum than women who delivered at term (severe preeclampsia, 24.1% [95% CI, 22.8%-25.4%] vs 20.9% [95% CI, 19.6%-22.1%]; preeclampsia, 16.2% [95% CI, 14.6%-17.8%] vs 10.8% [95% CI, 10.3%-11.3%]; GH, 20.6% [95% CI, 17.2%-24.0%] vs 11.6% [95% CI, 10.9%-12.3%]; no HDP, 2.3% [95% CI, 2.2%-2.4%] vs 1.7% [95% CI, 1.7%-1.7%]) (eTable 2 in Supplement 1). In contrast, similar proportions of women with or without small-for-gestational-age infants initiated medication use post partum (severe preeclampsia, 23.6% [95% CI, 22.2%-25.0%] vs 21.3% [95% CI, 20.1%-22.5%]; preeclampsia, 12.1% [95% CI, 11.1%-13.1%] vs 11.2% [95% CI, 10.7%-11.7%]; GH, 15.3% [95% CI, 13.5%-17.1%] vs 11.5% [95% CI, 10.8%-12.2%]; no HDP, 1.7% [95% CI, 1.6%-1.8%] vs 1.7% [95% CI, 1.7%-1.7%]) (eTable 2 in Supplement 1).

### Timing of Initiation of Postpartum Antihypertensive Medication Use

Most women with an HDP who initiated antihypertensive medication use post partum filled their first prescription within 3 months of delivery (severe preeclampsia, 86.6% [95% CI, 84.6%-89.4%]; preeclampsia, 75.3% [95% CI, 73.8%-76.2%]; and GH, 75.1% [95% CI, 72.9%-77.1%]). In comparison, 23.3% (95% CI, 22.5%-23.5%) of women without HDPs who initiated antihypertensive medication use did so within 3 months of delivery. However, 13.4% to 24.9% of women with an HDP (severe preeclampsia, 13.4% [95% CI, 11.9%-14.1%]; preeclampsia, 24.7.% [95% CI, 24.0%-26.0%]; GH, 24.9% [95% CI, 22.5%-27.5%]) first initiated antihypertensive medication use more than 3 months post partum, and 5.5% to 11.6% (severe preeclampsia, 6.5% [95% CI, 5.6%-7.4%]; preeclampsia, 5.5% [95% CI, 4.9%-6.1%]; GH, 11.6% [95% CI, 10.0%-13.2%]) filled their first prescription more than 1 year post partum. Compared with women without HDPs, women with HDPs were 23 to 57 times more likely to initiate antihypertensive medication use within 3 months of delivery (severe preeclampsia: HR, 56.9 [95% CI, 53.5-60.5]; preeclampsia: HR, 23.2 [95% CI, 21.9-27.7]; GH: HR, 23.2 [95% CI, 21.4-25.1]) (Table 2). Although HRs decreased thereafter, women with HDPs had rates of initiation of antihypertensive therapy up to 5 times higher than those observed among women without HDPs. For women with GH or severe preeclampsia, HRs increased in magnitude from 12 to 17 months to 18 to 23 months (severe preeclampsia: HR at 12-17 months, 1.84 [95% CI, 1.36-2.50]; HR at 18-23 months, 2.59 [95% CI, 1.99-3.36]; *P* < .001; preeclampsia: HR at 12-17 months, 2.23 [95% CI, 1.89-2.63]; HR at 18-23 months, 1.99 [95% CI, 1.67-2.38]; *P* = .22; GH: HR at 12-17 months, 1.94 [95% CI, 1.47-2.56]; HR at 18-23 months, 2.80 [95% CI, 2.22-3.53]; *P* < .001) (Table 2).

**Table 2.** Associations Between Type of HDP and Postpartum Initiation of Antihypertensive Medication Use, by Time Since Delivery After a Woman’s First Pregnancy, 1995-2018, Denmark

Type of HDP	Time between delivery and filling a first prescription for antihypertensive medication			
	No. of person-years (×10^3^)	No. of events	Incidence rate^a^	HR (95% CI)
At <3 mo				
None	185.8	2967	1.6	1 [Reference]
GH	2.0	800	40.0	23.2 (21.4-25.1)
PE^b^	4.6	1716	37.3	23.2 (21.9-27.7)
Severe PE^c^	1.7	1568	92.2	56.9 (53.5-60.6)
At 3-5 mo				
None	187.2	1509	0.8	1 [Reference]
GH	2.0	56	2.8	3.75 (2.89-4.91)
PE^b^	4.6	133	2.9	3.70 (3.10-4.42)
Severe PE^c^	1.6	61	3.8	4.81 (3.72-6.22)
At 6-11 mo				
None	364.3	2774	0.8	1 [Reference]
GH	3.9	83	2.1	3.12 (2.50-3.88)
PE^b^	8.9	153	1.7	2.32 (1.97-2.73)
Severe PE^c^	3.1	82	2.6	3.66 (2.94-4.57)
At 12-17 mo^d^				
None	344.5	2799	0.8	1 [Reference]
GH	3.7	52	1.4	1.94 (1.47-2.56)
PE^b^	8.4	148	1.8	2.23 (1.89-2.63)
Severe PE^c^	3.0	42	1.4	1.84 (1.36-2.50)
At 18-23 mo^d^				
None	310.4	2694	0.9	1 [Reference]
GH	3.3	74	2.2	2.80 (2.22-3.53)
PE^b^	76.1	128	1.7	1.99 (1.67-2.38)
Severe PE^c^	2.7	58	2.2	2.59 (1.99-3.36)

Abbreviations: GH, gestational hypertension; HDP, hypertensive disorder of pregnancy; HR, hazard ratio; PE, preeclampsia.

^a^ Incidence rate per 10 000 person-years.

^b^ Preeclampsia without severe features.

^c^ Preeclampsia with severe features.

^d^ Estimates from 12 to 17 months compared with from 18 to 23 months were significantly different for GH (*P* < .001) and severe preeclampsia (*P* < .001).

### Number of Filled Postpartum Prescriptions

**Figure 2.**
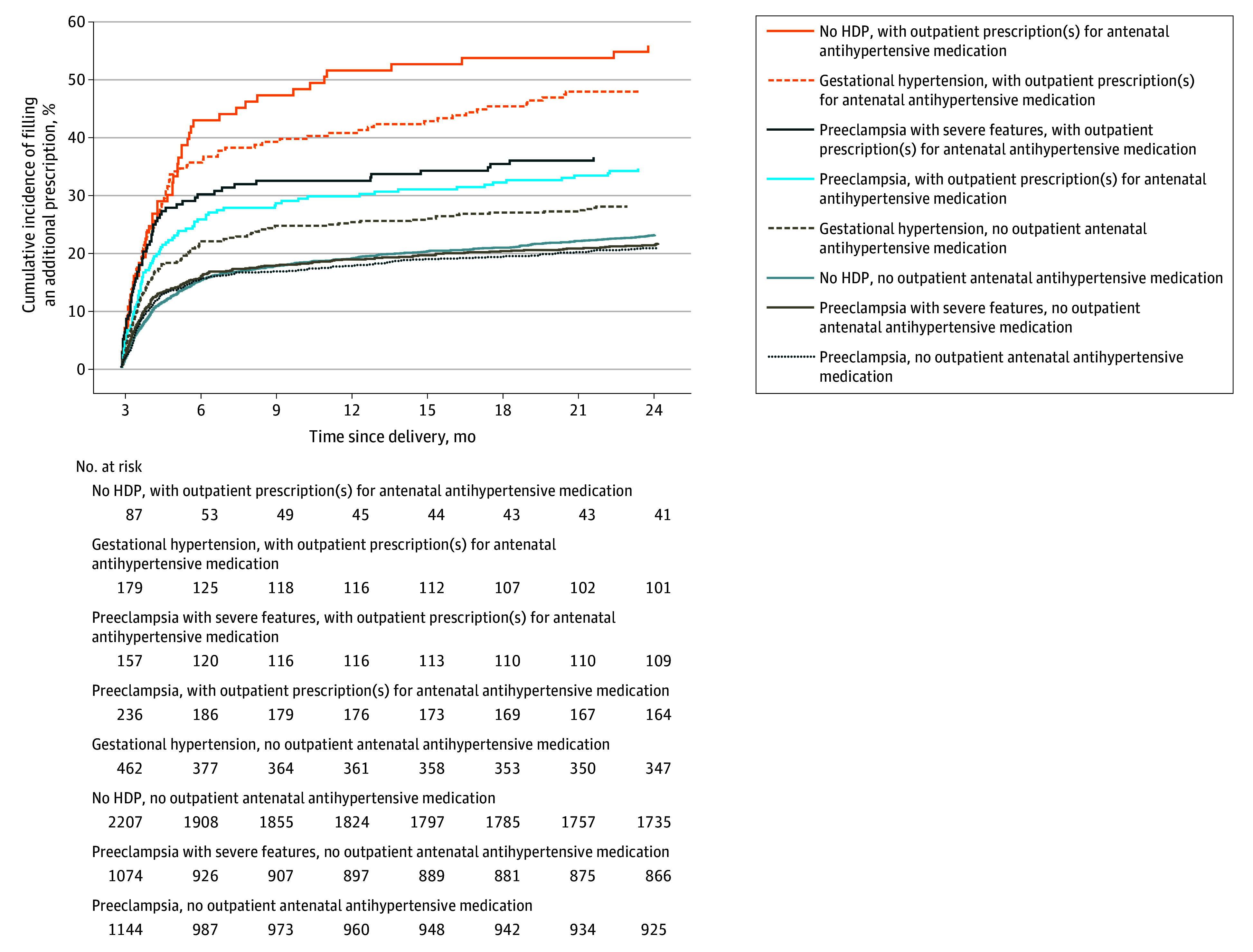
Two-Year Cumulative Incidences of Filling a Second Prescription for Antihypertensive Medication Among Women Who Had Filled a First Prescription Within 12 Weeks of Delivery, by Type of Hypertensive Disorder of Pregnancy (HDP) and Whether or Not Outpatient Prescriptions for Antihypertensive Medication Were Filled During Pregnancy, After a Woman’s First Delivery in the Study Period, 1995-2018, Denmark Preeclampsia with severe features includes eclampsia or the HELLP (hemolysis, elevated liver enzymes and low platelets) syndrome. A lack of antenatal outpatient prescriptions for antihypertensive medication does not preclude the receipt of inpatient or outpatient clinic–based treatment with antihypertensive medication during pregnancy, as medications dispensed by hospital pharmacies are not registered in the Danish National Prescription Register (which contains information only on prescriptions filled at community pharmacies).

Depending on HDP type and requirement for antenatal medication, 21.8% (95% CI, 19.5%-24.3%) to 55.9% (95% CI, 46.2%-66.1%) of women who filled a first postpartum prescription for antihypertensive medication within 12 weeks of delivery required another prescription more than 3 months after delivery (Figure 2; eTable 1 in Supplement 1). Women who had GH were more likely to fill additional prescriptions than women with other forms of HDP, with the majority of such women filling further prescriptions within 6 months of delivery (Figure 2; eTable 1 in Supplement 1).

### Findings in Pregnancies Without HDPs

Women without an HDP diagnosis who did not use antihypertensive medication during pregnancy had a 2-year cumulative incidence of postpartum initiation of medication use of 1.8% (95% CI, 1.8%-1.8%) (eTable 1 in Supplement 1); 23.3% (95% CI, 22.5%-23.5%) of these women initiated treatment within 3 months of delivery. Only 1324 of 747 822 women (0.2%) without an HDP diagnosis were prescribed antenatal antihypertensive medication, but their 2-year cumulative incidence of postpartum antihypertensive medication use (13.9% [95% CI, 11.9%-15.3%]) exceeded that of women with GH (10.0% [95% CI, 9.3%-10.7%]) or preeclampsia (10.4% [95% CI, 9.9%-10.8%]) without filled prescriptions for antenatal medication (Figure 1; eTable 1 in Supplement 1). Among women without an HDP diagnosis (with or without antenatal medication) who filled a postpartum prescription for antihypertensive medication, 76.7% (95% CI, 76.3%-77.1%) first did so more than 3 months after delivery and 43.1% (95% CI, 42.7%-43.5%) did so more than 1 year post partum. Women without a registered HDP diagnosis who had used antenatal medication were most likely to fill multiple prescriptions after initiation of postpartum medication use; 55.9% (95% CI, 46.2%-66.1%) required additional prescriptions for antihypertensive medication more than 3 months after delivery (Figure 2; eTable 1 in Supplement 1).

### Choice of Postpartum Antihypertensive Medication

**Figure 3.**
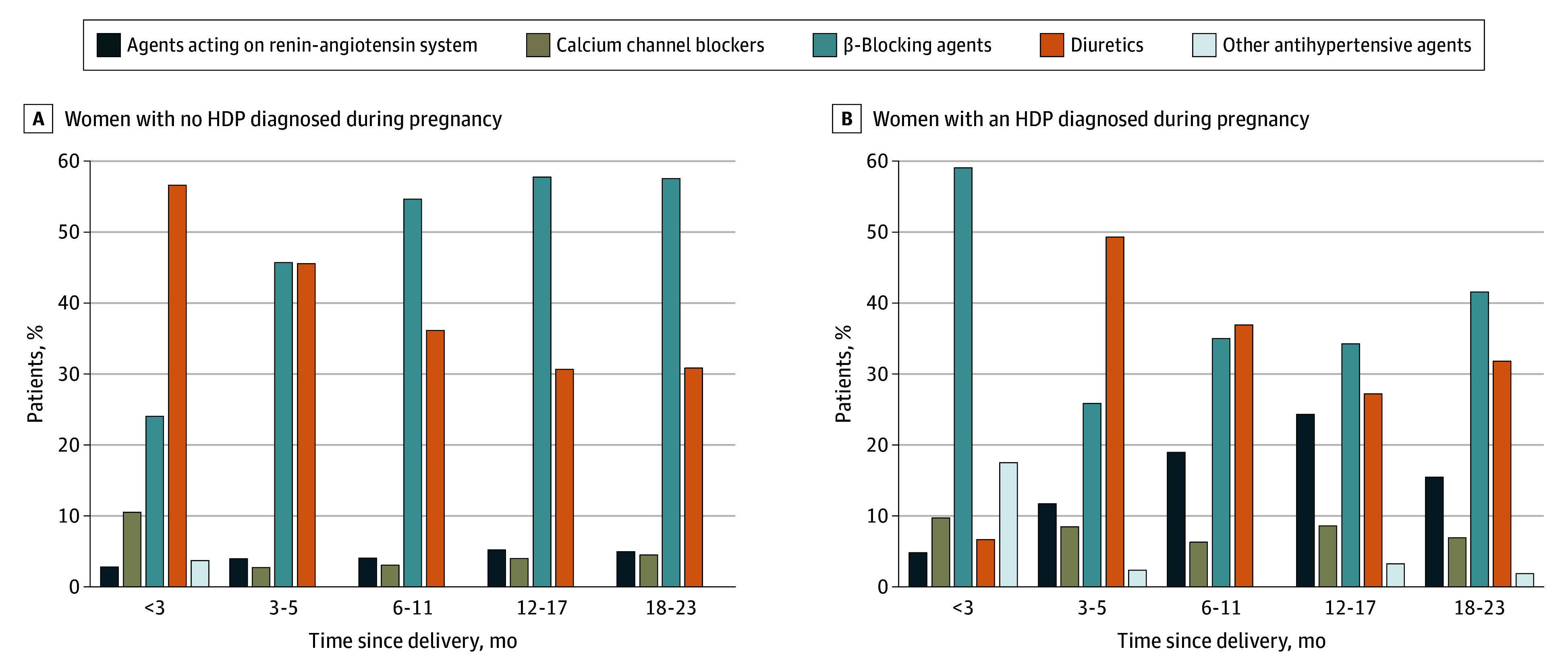
First Choice of Postpartum Antihypertensive Medication by Timing of Prescription Within 2 Years of Delivery Among Women Who Had and Did Not Have Hypertensive Disorders of Pregnancy (HDPs) in Their First Pregnancy in the Study Period, 1995-2018, Denmark

When we examined the maximum number of medications used in a 30-day period, overall, most (92.5% [95% CI, 92.2%-92.9%]) women filling postpartum prescriptions for antihypertensive medication used a single medication; 6.5% (95% CI, 6.1%-6.9%) used 2 medications, 0.8% (95% CI, 0.7%-0.9%) used 3 medications, and 0.1% (95% CI, 0.5%-0.15%) used 4 or more medications (eTable 3 in Supplement 1). However, more women with HDPs (up to 20.2% [95% CI, 19.1%-21.3%]) filled prescriptions for more than 1 medication in a 30-day period. The type of medication prescribed depended on HDP status and timing of postpartum initiation of use (Figure 3). Generally, β-blocking agents and diuretics were the first-line medications during the study period, followed by renin-angiotensin system blockers, mostly for women with HDPs. Calcium channel blockers and other antihypertensive medications were rarely used as first-line therapies. Over the 23-year study period, use of diuretics gradually decreased, while use of calcium channel blockers and renin-angiotensin system blockers increased, particularly after 2010 (eFigure in Supplement 1).

## Discussion

In a large population-based cohort of women with pregnancies between 1995 and 2018, up to 44.1% of women who had HDPs initiated antihypertensive medication use within 2 years of delivery, with most doing so within 3 months of delivery. Women with HDPs were up to 57 times more likely than women without registered HDPs to fill a first prescription for antihypertensive medication in the first 3 months post partum, with the highest medication initiation rates observed among women who had had severe preeclampsia. Among women who initiated use of antihypertensive medication within 3 months of delivery, up to 55.9% filled additional prescriptions thereafter. Although β-blocking agents and diuretics were the most widely used antihypertensive agents, choice of medication varied by year of prescription, HDP diagnosis, and timing of initiation of use.

### Implications of Study Findings for Postpartum Blood Pressure Control Policy

Approximately 40% of women with HDPs may require antihypertensive therapy within 6 weeks of delivery, emphasizing the need for postpartum follow-up in this group of women.^20^ The American College of Obstetricians and Gynecologists recently recommended that after an HDP, women should have their blood pressure evaluated no later than 7 to 10 days post partum, with additional monitoring tailored to each woman’s individual needs.^21,22^ They also highlighted the “fourth-trimester” care gap that exists because it is unclear which specialty (obstetrics, family practice, cardiology) should be responsible for postpartum follow-up for these women. The United Kingdom’s National Institute for Health and Care Excellence also recommends blood pressure monitoring for at least 14 days post partum for women with preeclampsia, without specifying whether further monitoring is warranted or who bears the responsibility for such monitoring.^23^

Tight blood pressure control in the immediate postpartum period produces lasting reductions in blood pressure,^12,13^ potentially reducing the risk of long-term cardiovascular disease among women who had HDPs by more than 30%.^10,11,14,24,25^ However, among women with HDPs in our cohort who initiated use of antihypertensive medication post partum, up to one-fourth did so more than 3 months after delivery, suggesting that a considerable proportion of these women may have unrecognized or untreated hypertension in the immediate postpartum period. A delay in recognition and effective management might explain why women who had HDPs exhibited marked increases in initiation of antihypertensive therapies more than 18 months post partum, compared with women without HDPs.

The problem of unrecognized and undertreated hypertension among postpartum women is unlikely to be specific to Denmark and may be even more pronounced in settings without free universal health care. Moreover, women with GH typically receive less postpartum attention than women with preeclampsia, although our results suggest that the former were more likely to require additional antihypertensive medication after an initial postpartum prescription. The natural history of postpartum blood pressure after HDPs is poorly studied, but 1 small study showed that 50% of women who had preeclampsia still had hypertension 6 to 12 weeks post partum, both overall and among women still receiving treatment.^14^ The lack of robust data does not detract from the importance of early diagnosis and effective management of persistent postpartum hypertension as a strategy to reduce long-term cardiovascular disease among these women. In fact, the benefits of postpartum clinics offering standardized care to women at greatest risk of long-term adverse outcomes after pregnancies complicated by HDPs are currently being evaluated.^26,27,28^ Future research should evaluate the clinical efficacy of systematic postpartum blood pressure monitoring and early management of postpartum hypertension in preventing diagnostic delays, decreasing the burden of chronic hypertension, and improving cardiovascular disease prevention among women after HDPs.

### Choice of Antihypertensive Medication

Overall, women who had HDPs were most frequently first prescribed β-blocking agents post partum, possibly because they simply continued with the medication they used antenatally, when β-blockers are often used. However, consistent with recent recommendations that renin-angiotensin system blockers be the first-line postpartum antihypertensive treatment in women who had HDPs (except in women of African or Caribbean origin, among whom calcium channel blockers are the preferred first-line agents),^23^ we observed a shift toward renin-angiotensin system blocker use later in the study period among these women. In contrast, women with neither an HDP diagnosis nor antenatal antihypertensive medication use were primarily prescribed diuretics post partum.

### Strengths and Limitations

This study has some strengths. Our cohort’s size allowed us to produce a detailed description of the initiation of antihypertensive medication use in the immediate postpartum period by HDP diagnosis and time since delivery. The use of prospectively collected registry data from the entire Danish population minimized the risk of selection bias and eliminated the possibility of recall bias. Hypertensive disorders of pregnancy diagnoses in the National Patient Register have been shown to have a specificity greater than 99%,^29^ indicating that most registered diagnoses are correct. However, the register’s sensitivity for HDPs was only 69% for preeclampsia and 10% for GH, indicating that many women, particularly those with GH, may not be registered as having had an HDP, and women with the most severe cases of hypertension in pregnancy may be overrepresented in our HDP groups, making it difficult to generalize our results to women with milder disease. On the other hand, women with milder disease (GH in particular) who were managed on an outpatient basis may have comprised most of the women with no HDP diagnosis who filled prescriptions for antihypertensive medication during pregnancy. This group likely also included some women with either undiagnosed or unregistered preexisting chronic hypertension. However, because the prevalence of chronic hypertension among women of childbearing age in the general population is low, because our cohort included all Danish residents with pregnancies in the study period, and because we excluded all women who filled prescriptions for antihypertensive medication before pregnancy, women with unregistered chronic hypertension were likely only a minority of the group of women without a registered HDP diagnosis who filled prescriptions for antenatal antihypertensive medication.

This study also has some limitations. The National Prescription Registry contains information only on prescriptions filled at community pharmacies. Medications administered in the hospital or provided by hospital-based outpatient clinics are not registered,^19^ which explains the low proportion of women with HDPs who received antenatal medication (10.5%). Some women receiving postpartum medication before discharge might therefore also have been misclassified as not using postpartum antihypertensive medication, but most would have been discharged with an additional prescription for antihypertensive medication, making their identification inevitable, if potentially delayed.

The Danish registries do not include information on postpartum blood pressure measurements. Consequently, the study population included women for whom antihypertensive medication was not indicated; we report the incidence of postpartum initiation of medication use among all women who delivered in the study period rather than only among women who continued to have hypertension post partum. Therefore, our results potentially underestimate the proportion of women receiving correct post-HDP follow-up with respect to postpartum blood pressure. Information on factors associated with physician prescribing practices (eg, specialty, experience, academic affiliation, health care sector, regional socioeconomic factors) and patient care-seeking behavior (eg, income and educational level) would have provided further nuance to our findings but was unavailable to us. Despite these limitations, the observed patterns of initiation of medication use indicate that for a significant fraction of women, identification and treatment of persistent postpartum hypertension may not occur promptly enough to reduce the risk of later cardiovascular disease, even in a country where all women have good access to care in the postpartum period.

## Conclusions

In this cohort study of 784 782 women, after an HDP, up to 44.1% of women initiated use of antihypertensive medication within 2 years after delivery, with up to one-fourth initiating medication use more than 3 months post partum. Increased awareness among affected women and health care professionals of the high risk of chronic hypertension after an HDP and the importance of timely ascertainment and effective management of persistent postpartum hypertension are vital to reducing the cardiovascular disease risks associated with HDPs. Future studies of how to deliver such care with the greatest effect are essential.

## Notes

Supplement 1.
eAppendix.
**eTable 1.** Cumulative Incidences of Filling New Prescriptions for Antihypertensive Medication After the First Delivery in the Study Period, by History of Hypertensive Disorder of Pregnancy, Denmark 1995-2018 **eTable 2.** Proportion of Women Initiating Use of Antihypertensive Medication Within 2 Years of Their First Delivery in the Study Period, by Hypertensive Disorder of Pregnancy, Gestational Age at Delivery, and Infant Birthweight for Gestational Age, Denmark, 1995-2018 **eTable 3.** Maximum Number of Antihypertensive Medications Used Within 30 Days for Women Filling Postpartum Prescriptions Within Two Years of Their First Delivery in 1995-2018, Denmark **eFigure.** First Choice of Postpartum Antihypertensive Medication Within Two Years of Delivery After a First Pregnancy, by Calendar Year, in 1995-2018, Denmark
eReference.

Supplement 2.
Data Sharing Statement
