# Supplementary material for: Patterns of Antihypertensive Medication Use in the First 2 Years Post Partum
Source: JAMA Netw Open. 2024 Aug 7;7(8):e2426394. doi: 10.1001/jamanetworkopen.2024.26394 (PMC11307130; doi:10.1001/jamanetworkopen.2024.26394)
Supplement: Supplement 1. — eAppendix. eTable 1. Cumulative Incidences of Filling New Prescriptions for Antihypertensive Medication After the First Delivery in the Study Period, by History of Hypertensive Disorder of Pregnancy, Denmark 1995-2018 eTable 2. Proportion of Women Initiating Use of Antihypertensive Medication Within 2 Years of Their First Delivery in the Study Period, by Hypertensive Disorder of Pregnancy, Gestational Age at Delivery, and Infant Birthweight for Gestational Age, Denmark, 1995-2018 eTable 3. Maximum Number of Antihypertensive Medications Used Within 30 Days for Women Filling Postpartum Prescriptions Within Two Years of Their First Delivery in 1995-2018, Denmark eFigure. First Choice of Postpartum Antihypertensive Medication Within Two Years of Delivery After a First Pregnancy, by Calendar Year, in 1995-2018, Denmark eReference. [file jamanetwopen-e2426394-s001.pdf]

Supplementary Online Content

Lihme F, Basit S, Thilaganathan B, Boyd HA. Patterns of antihypertensive medication use in the first 2 years post partum. *JAMA Netw Open.* 2024;7(8):e2426394. doi:10.1001/jamanetworkopen.2024.26394

eAppendix.
eTable 1. Cumulative Incidences of Filling New Prescriptions for Antihypertensive Medication After the First Delivery in the Study Period, by History of Hypertensive Disorder of Pregnancy, Denmark 1995-2018
eTable 2. Proportion of Women Initiating Use of Antihypertensive Medication Within 2 Years of Their First Delivery in the Study Period, by Hypertensive Disorder of Pregnancy, Gestational Age at Delivery, and Infant Birthweight for Gestational Age, Denmark, 1995-2018
eTable 3. Maximum Number of Antihypertensive Medications Used Within 30 Days for Women Filling Postpartum Prescriptions Within Two Years of Their First Delivery in 1995-2018, Denmark
eFigure. First Choice of Postpartum Antihypertensive Medication Within Two Years of Delivery After a First Pregnancy, by Calendar Year, in 1995-2018, Denmark
eReference.

This supplementary material has been provided by the authors to give readers additional information about their work.

© 2024 Lihme F et al. *JAMA Network Open.*

eAppendix.

Exclusions in the study cohort, definitions:

- Women with cardiovascular disease registered before their first pregnancy in the study period (International Classification of Diseases, eighth revision [ICD-8], codes 390-444 or tenth revision [ICD-10] codes I00-I77)
- Women with hypertension diagnosed or treated before week 20 of their first pregnancy in the study period (ICD-8 codes 400-404 or ICD-10 codes I10-I15 or O10-O11 registered in the National Patient Registry, or the filling of two or more prescriptions for antihypertensive medication [Anatomic Therapeutic Chemical {ATC} codes C02, C03, C07-C09] in the six months before conception or of one prescription between conception and 20 weeks' gestation).

Definition of hypertensive disorders of pregnancy (HDP):

- Gestational hypertension: ICD-8 code 637.00, ICD-10 codes O13.9 or O16.9)
- Preeclampsia without severe features: ICD-8 codes 637.03, 637.09, or 637.99, ICD-10 codes O14.0 or O14.9
- Preeclampsia with severe features, including the HELLP syndrome and eclampsia: ICD-8 codes 637.04, 637.19, 762.19, 762.29, or 762.39, ICD-10 codes O14.1, O14.2, or O15.0-15.9.

Women were considered to have a diagnosis of a hypertensive disorder of pregnancy, if they were registered in National Patient Register with one of the above ICD codes at any time between one month before delivery and seven days postpartum. Because diagnoses are re-registered at every contact with obstetric care, women whose only diagnoses were registered outside this time window were not considered to have an HDP to minimize misclassification of women in whom suspected HDPs could not be confirmed as having an HDP. Since by definition an HDP involves

© 2024 Lihme F et al. *JAMA Network Open.*

new-onset hypertension with or without other signs of organ dysfunction developing ≥20 weeks gestation, we considered only women with diagnoses registered after this point to have an HDP. If multiple diagnoses were registered, the woman was assigned the most severe diagnosis registered during the pregnancy.

<u>Postpartum use of antihypertensive medication, definitions:</u>

Anatomic Therapeutic Chemical (ATC) codes:

- Beta blocking agents: C07
- Calcium channel blockers: C08
- Agents acting on the renin–angiotensin system: C09
- Diuretics: C03
- Other specified antihypertensive agents: C02

Combination medications were classified according to the component prioritized by the ATC code under which they were registered.

© 2024 Lihme F et al. *JAMA Network Open.*

eTable 1. Cumulative Incidences of Filling New Prescriptions for Antihypertensive Medication After the First Delivery in the Study Period, by History of Hypertensive Disorder of Pregnancy, Denmark 1995-2018

Cumulative incidence of:	HDP diagnosis during pregnancy	Anti-hypertensive medication prescribed during pregnancy	Cumulative incidence (%) (95% CI)
Filling a first prescription within two years of delivery	None	Yes	13.9 (11.9-15.3)
		No	1.8 (1.8-1.8)
	Gestational hypertension	Yes	37.5 (34.1-41.1)
		No	10.0 (9.3-10.7)
	Preeclampsia[a]	Yes	32.2 (29.5-35.1)
		No	10.4 (9.9-10.8)
	Severe preeclampsia[b]	Yes	44.1 (40.0-48.2)
		No	21.0 (20.1-22.0)
Filling a first prescription within 3 months of delivery	None	Yes	8.4 (7.1-9.9)
		No	0.4 (0.4-0.5)
	Gestational hypertension	Yes	31.3 (28.1-34.7)
		No	7.5 (7.0-8.0)
	Preeclampsia[a]	Yes	28.5 (25.9-31.3)
		No	7.8 (7.3-8.1)
	Severe preeclampsia[b]	Yes	38.3 (34.4-42.5)
		No	18.5 (17.6-19.3)
Filling a further prescription after 3 months[c]	None	Yes	55.9 (46.2-66.1)
		No	23.2 (21.5-25.0)
	Gestational hypertension	Yes	48.4 (41.7-55.7)
		No	28.3 (24.5-32.5)
	Preeclampsia[a]	Yes	34.7 (29.1-40.9)
		No	21.0 (19.5-24.3)
	Severe preeclampsia[b]	Yes	36.6 (29.9-44.3)
		No	21.8 (19.5-24.3)

HDP, hypertensive disorder of pregnancy; CI, Confidence Interval
[a]Preeclampsia without severe features
[b]Preeclampsia with severe features
[c]Among women who filled a first prescription within 12 weeks of delivery, 2-year cumulative incidence

© 2024 Lihme F et al. *JAMA Network Open.*

eTable 2. Proportion of Women Initiating Use of Antihypertensive Medication Within 2 Years of Their First Delivery in the Study Period, by Hypertensive Disorder of Pregnancy, Gestational Age at Delivery, and Infant Birthweight for Gestational Age, Denmark, 1995-2018

	Hypertensive disorder of pregnancy	Use of postpartum antihypertensive medication, N (%)<br>Yes<br>N=17,897	 No<br>N=766,885
Gestational age at delivery			
		N (%, 95% CI)	N (%, 95% CI)
<37 weeks	Severe preeclampsia[a]	937 (24.1, 22.8-25.4)	2,958 (75.9, 74.6-77.2)
	Preeclampsia[b]	344 (16.2, 14.6-17.8)	1,774 (83.8, 82.2-85.4)
	Gestational hypertension	112 (20.6, 17.2-24.0)	431 (79.4,76.0-82.8)
	None	1,018 (2.3, 2.2-2.4)	44,050 (97.7, 97.6-97.8)
≥37 weeks	Severe preeclampsia	862 (20.9, 19.6-22.1)	3,264 (79.1, 77.9-80.3)
	Preeclampsia	1,911 (10.8, 10.3-11.3)	15,861 (89.3, 88.9-89.8)
	Gestational hypertension	948 (11.6, 10.9-12.3)	7,241 (88.4, 87.7-89.1)
	None	11,526 (1.7, 1.67-1.73)	679,056 (98.3, 98.27-98.33)
Unknown§	Severe preeclampsia	12 (21.4, 10.7-32.1)	44 (78.6, 67.9-89.3)
	Preeclampsia	23 (14.3, 8.9-19.7)	138 (85.7, 80.3-91.1)
	Gestational hypertension	5 (12.5, 2.3-22.8)	35 (87.5, 77.3-97-8)
	None	199 (1.6, 1.4-1.8)	12,037 (98.4, 98.2-98.6)
Small-for-gestational age infant[c]			
Yes	Severe preeclampsia	789 (23.6, 22.2-25.0)	2,549 (76.4, 75.0-77.8)
	Preeclampsia	501 (12.1, 11.1-13.1)	3,636 (87.9, 86.9-88.9)
	Gestational hypertension	243 (15.3, 13.5-17.1)	1,342 (84.7, 82.9-86.5)
	None	1,662 (1.7, 1.6-1.8)	94,761 (98.3, 98.2-98.4)
No	Severe preeclampsia	984 (21.3, 20.1-22.5)	3,626 (78.7, 77.5-79.9)
	Preeclampsia	1,756 (11.2, 10.7-11.7)	13,982 (88.8, 88.3-89.3)
	Gestational hypertension	818 (11.5, 10.8-12.2)	6,308 (88.5, 87.8-89.2)
	None	10,914 (1.7, 1.67-1.73)	629,298 (98.3, 98.27-98.33)
Unknown[d]	Severe preeclampsia	38 (29.5, 21.6-37.4)	91 (70.5, 62.6-78.4)
	Preeclampsia	21 (12.0, 7.2-16.8)	155 (88.1, 83.2-92.8)
	Gestational hypertension	<5	*
	None	167 (1.5, 1.3-1.7)	11,080 (98.5,98.3-98.7)

N, Number. CI, Confidence Interval

* To avoid potential identification of persons in categories with numbers <5, some numbers will be hidden.

[a] Preeclampsia with severe features

[b] Preeclampsia without severe features

[c]Birthweight for gestational week and sex <10th percentile according to a Scandinavian reference population[1]

[d]Gestational age at delivery unknown

© 2024 Lihme F et al. *JAMA Network Open.*

eTable 3. Maximum Number of Antihypertensive Medications Used Within 30 Days for Women Filling Postpartum Prescriptions Within Two Years of Their First Delivery in 1995-2018, Denmark

Maximum no. of medications used in a 30-day period	Number (%, 95% Confidence Interval)			
	No hypertensive disorder of pregnancy	Gestational hypertension	Preeclampsia[a]	Severe Preeclampsia[b]
1	12,257 (96.2,95.9-96.5)	872 (82.1, 79.8-84.4)	1,989 (87.3, 85.9-88.7)	1,445 (79.8, 78.0-81.6)
2	420 (3.3, 3.0-3.6)	162 (15.2, 13.0-17.4)	256 (11.2, 9.9-12.5)	321 (17.7, 15.9-19.5)
3	61 (0.5, 0.4-0.6)	24 (2.3, 1.4-3.2)	*	*
≥ 4	5 (0.04, 0.005-0.073)	7 (0.1, -0.1-0.3)	<5	<5

* To avoid potential identification of persons in categories with numbers <5, some numbers will be hidden.
[a] Preeclampsia without severe features
[b] Preeclampsia with severe features

© 2024 Lihme F et al. *JAMA Network Open.*

eFigure. First Choice of Postpartum Antihypertensive Medication Within Two Years of Delivery After a First Pregnancy, by Calendar Year, in 1995-2018, Denmark

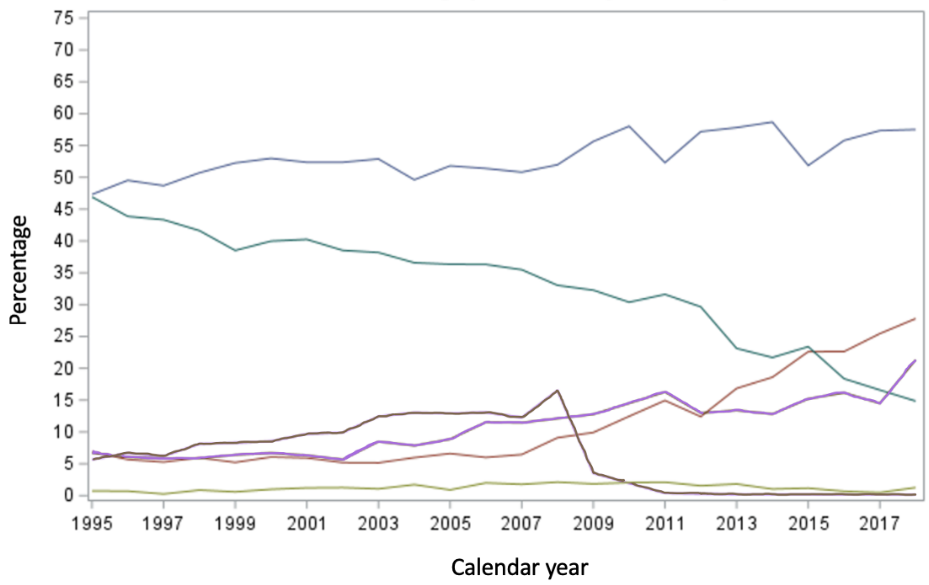

Red, calcium channel blockers (C08). Dark green: diuretics (C03). Purple, agents acting on the renin-angiotensin system (C09). Blue: beta-blocking agents (C07). Brown: other specified antihypertensive agents (C02). Light green: combination drugs (C02L, C02N, C07B, C07D, C09B, C09D).

© 2024 Lihme F et al. *JAMA Network Open.*

eReference.

1 Maršál K, Persson PH, Larsen T, Lilja H, Selbing A, Sultan B. Intrauterine growth curves based on ultrasonically estimated foetal weights. *Acta Paediatrica, International Journal of Paediatrics* 1996; **85**: 843–848.

© 2024 Lihme F et al. *JAMA Network Open.*
