# Supplementary material for: Patterns of Antihypertensive Medication Use in the First 2 Years Post Partum
Source: JAMA Netw Open. 2024 Aug 7;7(8):e2426394. doi: 10.1001/jamanetworkopen.2024.26394 (PMC11307130; doi:10.1001/jamanetworkopen.2024.26394)
Supplement: Supplement 2. — Data Sharing Statement [file jamanetwopen-e2426394-s002.pdf]

Data Sharing Statement

Lihme. Patterns of Antihypertensive Medication Use in the First 2 Years Post Partum. *JAMA Netw Open*. Published August 07, 2024. doi:10.1001/jamanetworkopen.2024.26394

Data
Data available: No

Additional Information
Explanation for why data not available: The data used in this study were retrieved from the Danish national registers and do not belong to the authors but to the Danish Health Data Authority. The authors are therefore not permitted to make the data publicly available. However, upon receipt of approval from the Danish Data Protection Agency, data from the Danish national health registers are available upon application through an online request system that can be found on the Danish Health Data Authority's website (https://sundhedsdatastyrelsen.dk/da/registre-og-services).
